# Simultaneous absolute quantification and sequencing of fish environmental DNA in a mesocosm by quantitative sequencing technique

**DOI:** 10.1038/s41598-021-83318-6

**Published:** 2021-02-23

**Authors:** Tatsuhiko Hoshino, Ryohei Nakao, Hideyuki Doi, Toshifumi Minamoto

**Affiliations:** 1 Kochi Institute for Core Sample Research, Japan Agency for Marine-Earth Science and Technology, Kochi, Japan; 2 Graduate School of Science and Technology for Innovation, Yamaguchi University, Yamaguchi, Japan; 3 Graduate School of Human Development and Environment, Kobe University, Kobe, Japan; 4 Graduate School of Simulation Studies, University of Hyogo, Kobe, Japan

**Keywords:** Targeted resequencing, Freshwater ecology

## Abstract

The combination of high-throughput sequencing technology and environmental DNA (eDNA) analysis has the potential to be a powerful tool for comprehensive, non-invasive monitoring of species in the environment. To understand the correlation between the abundance of eDNA and that of species in natural environments, we have to obtain quantitative eDNA data, usually via individual assays for each species. The recently developed quantitative sequencing (qSeq) technique enables simultaneous phylogenetic identification and quantification of individual species by counting random tags added to the 5′ end of the target sequence during the first DNA synthesis. Here, we applied qSeq to eDNA analysis to test its effectiveness in biodiversity monitoring. eDNA was extracted from water samples taken over 4 days from aquaria containing five fish species (*Hemigrammocypris neglectus*, *Candidia temminckii*, *Oryzias latipes*, *Rhinogobius flumineus*, and *Misgurnus anguillicaudatus*), and quantified by qSeq and microfluidic digital PCR (dPCR) using a TaqMan probe. The eDNA abundance quantified by qSeq was consistent with that quantified by dPCR for each fish species at each sampling time. The correlation coefficients between qSeq and dPCR were 0.643, 0.859, and 0.786 for *H. neglectus*, *O. latipes*, and *M. anguillicaudatus*, respectively, indicating that qSeq accurately quantifies fish eDNA.

## Introduction

Investigating biodiversity, population size, and time-course changes associated with environmental change is important for the conservation of biodiversity. Conventionally, population monitoring in the natural environment has required experts to classify and count species. Although the direct monitoring of species is a reliable approach, it is laborious and relies on intense monitoring and sampling efforts. Moreover, certain species, such as nocturnal species, are difficult to investigate, and direct monitoring is sometimes invasive to the environment and individual organisms.

Environmental DNA (eDNA) is a mixture of DNA released into the environment from many different species in the form of mucus, saliva, faeces, urine, gametes, and skin^1–3^. The use of eDNA offers the potential to monitor species present in nature without sampling effort or visual identification expertise. In the past decade, eDNA has been used to investigate the biodiversity of various species including fish, plants, fungi, birds, and mammals^4^. The applications of eDNA analysis are not limited to the present environment, but also include studies of past biodiversity in ice cores and sediments^5,6^.

For ecological community eDNA analysis, marker genes (e.g. mtDNA and nuclear rRNA genes) used for taxonomic identification are amplified by PCR followed by high-throughput sequencing (HTS). Species abundances in the community are often determined according to the relative abundance of the sequences of each taxon in a sequence library. However, the quantification accuracy of these methods is compromised by various factors that can affect the PCR efficiency, such as the amplified DNA sequences (e.g. GC content or the base adjacent to primers) and primer sequences^7–10^.

In recent years, there have been growing expectations for the application of environmental DNA data from each species in the natural environment to estimate species biomass/abundance and monitor the effects of environmental destruction. To apply environmental DNA to the monitoring of species in the natural environment, it is very important to understand the correlation between quantitative environmental DNA data and species abundance. DNA quantification has been conventionally performed by quantitative PCR (qPCR)^11,12^ and more recently by digital PCR (dPCR), which is more accurate than the former because of tolerance to PCR inhibitory substances^13^. Quantification by qPCR or dPCR requires an assay to be established for each target, including the design of PCR primers, preparation of standards (if qPCR is used), and optimisation of PCR conditions. Therefore, parallel quantitative analysis of numerous species is not straightforward, although it has been recently attempted^14^.

**Figure 1:**
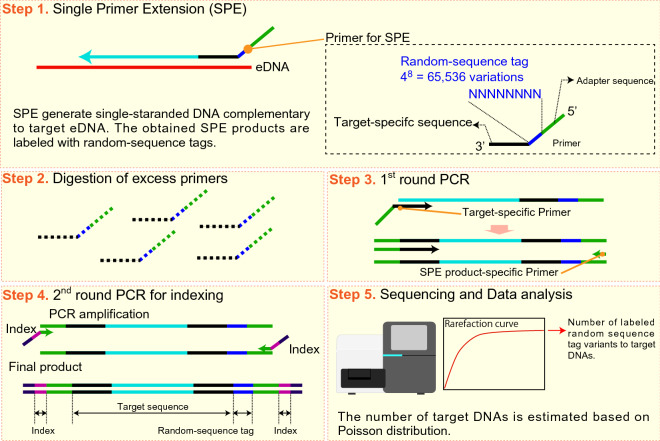
Schematic of quantitative sequencing (qSeq) procedure.

The recently developed quantitative sequencing (qSeq) technique^15,16^ enables simultaneous sequencing and quantification of DNA from many species in a single HTS run (Fig. 1). In qSeq analysis, a random sequence tag is added to the 5′ end of the target sequence during single primer extension (SPE) prior to PCR amplification to prepare the sequence library. If the variety of random tag sequences is sufficiently large relative to the number of targeting DNA molecules, the distribution of the random tag to DNA molecules follows the Poisson statistic^17^. Therefore, after HTS, the number of DNA molecules in a sample can be estimated by counting the variety of random tags at the 5′ end of the targeted sequence with minimal effect from PCR bias.

In this study, we applied qSeq to eDNA extracted from aquarium experiments comprising five fish species. The qSeq results were compared with the quantitative values obtained by dPCR to demonstrate the potential of qSeq for the comprehensive quantitative analysis of eDNA from natural environments.

## Methods

### Aquarium experiment and sampling

**Figure 2:**
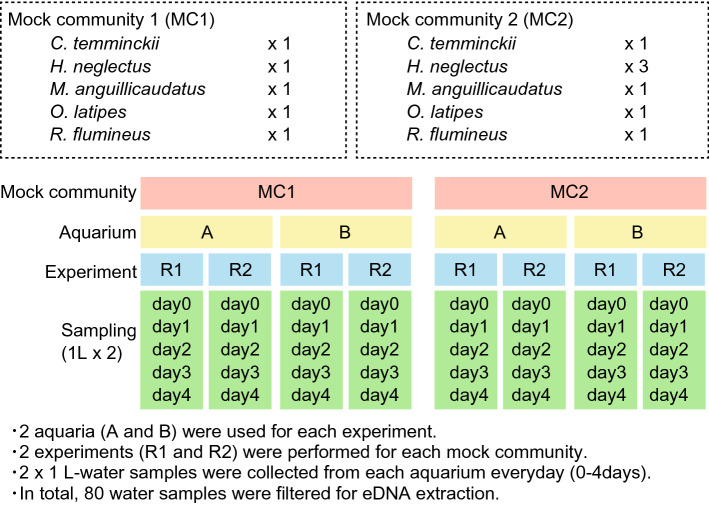
Experimental setup of the aquarium experiments.

To examine the effect of changes in species composition on the behaviour of eDNA, we conducted aquarium experiments using two mock fish communities comprising *H. neglectus*, *C. temminckii*, *O. latipes*, *R. flumineus*, and *M. anguillicaudatus*. Mock community 1 (MC1) consisted of one individual of each of the five fish species, whereas mock community 2 (MC2) consisted of three *H. neglectus* individuals and one individual of each of the other four fish species (Fig. 2). We used two aquaria (A and B). Each aquarium was used four times, twice for each mock community, giving two replicates (R1 and R2). This resulted in eight experimental units (2 mock fish communities × 2 aquaria × 2 replicates). Figure 2 shows the experimental setup used in this study.

To set up the aquaria, 20 L of tap water was added into each aquarium (GEX Co. Ltd., Osaka, Japan) and heated with a heater (Spectrum Brands, Wisconsin, US) until the water temperature reached 25 °C. Water in the two aquaria was maintained at 25 °C and constantly circulated with an aeration device. Before adding fish to the aquaria, the water was sampled for the negative control. The first experimental samples (day 0) were taken 1 h after adding the fish and subsequent samples were taken each day until day 4. At each sampling, two 1-L samples of surface water were collected from each aquarium and then 2 L of tap water was added to each aquarium to maintain the volume of water. The weight of individual fish species was measured using an electronic balance immediately after the final sampling. After each experiment, the two aquaria were bleached before being reused.

In Japan, experiments on fish do not require any legal procedures or permission. However, in order to avoid causing pain to the specimens, the experiments in this study were conducted in accordance with the ARRIVE guidelines, Japanese laws and guidelines for mammals, birds, and reptiles as below; Act on Welfare and Management of Animals (Notice of the Ministry of the Environment No. 105 of October 1, 1973), Standards relating to the Care and Keeping and Reducing Pain of Laboratory Animals (Notice of the Ministry of the Environment No. 88 of 2006), Fundamental Guidelines for Proper Conduct of Animal Experiment and Related Activities in Academic Research Institutions under the jurisdiction of the Ministry of Education (Notice of Ministry of Education No. 71, 2006), and Guidelines for Proper Conduct of Animal Experiments (established by the Science Council of Japan on June 1, 2006).

### DNA extraction

Each 1-L water sample was filtered immediately through a GF/F glass fibre filter (nominal pore size = 0.7 μm, diameter = 47 mm; GE Healthcare Japan Corporation, Tokyo, Japan). Filter funnels and measuring cups were bleached after filtration to prevent cross-contamination among the water samples. All filters were stored separately at − 20 °C until DNA extraction. Total eDNA was extracted from each filter using a DNeasy Blood and Tissue Kit (QIAGEN, Hilden, Germany) and Salivette tubes (Sarstedt AG & Co. KG, Nümbrecht, Germany). Extraction methods were as previously described^18^ with modifications. A filter sample was placed in the upper part of the Salivette tube and 220 μL of solution containing Buffer AL (200 μL) and Proteinase K (20 μL) was added. The tube containing the filter was incubated at 56 °C for 30 min, then centrifuged at 5000 × *g* for 3 min, and the solution was collected in the base of the tube. To increase eDNA yield, 220 μL Tris-EDTA (TE) buffer was added to the filter sample and centrifuged at 5000 × *g* for 1 min. Then, ethanol (200 μL) was added to the collected solution, and the mixture was transferred to a spin column. Total eDNA was eluted in buffer AE (100 μL), following the manufacturer’s instructions. All eDNA samples were stored at − 20 °C prior to qSeq and dPCR.

### Quantitative sequencing

Simultaneous quantification and sequencing of the extracted eDNA were performed by qSeq as previously described^15,16^. First, SPE was performed. The SPE reaction mixture (20 µL) consisted of 1 × PrimeSTAR Max premix (Takara Bio Inc., Kusatsu, Japan), 300 nM of the primer qSeq-MiFish-U-F (Table 1), and extracted DNA (2 µL). The SPE primer qSeq-MiFish-U-F contains an eight-base length random sequence tag, which creates 65,536 different variations, enabling the quantification of up to approximately 1.0 × 10^5^ copies of DNA^15^. This amount of variation was sufficient to quantify the abundance of eDNA in this study. SPE was initiated by denaturation at 94 °C for 1 min, followed by cooling to 60 °C at 0.3 °C/s, incubation at 60 °C for 1 min, and final extension at 70 °C for 10 min. Subsequently, the excess primer was completely digested by adding exonuclease I (4 µL, 5 U/µL; Takara Bio Inc.) to the SPE mixture. The digestion was performed at 37 °C for 120 min, followed by inactivation of the exonuclease I at 80 °C for 30 min. The first-round PCR mixture (25 µL) contained PrimeSTAR Max premix (12.5 µL), primers qSeq-MiFish-U-R and F2 (300 nM each; Table 1), and the SPE product (2 µL). Following 40 cycles of amplification at 98 °C for 10 s, 55 °C for 5 s, and 72 °C for 5 s, the amplification product was subjected to agarose gel electrophoresis, and the band of the expected size was removed and purified using Nucleospin Gel and PCR Clean-up column (Takara Bio Inc.). The qSeq-MiFish-U-R primer also contains eight N bases to increase the complexity, which improves the sequencing quality, and thus PhiX was not added in this study. Finally, a 2nd-round PCR was performed to add an index for Illumina sequencing as described elsewhere^15^. The indexed PCR amplicon was purified using AMPure XP beads (Beckman Coulter, Indianapolis, IN) followed by sequencing using a MiSeq platform with MiSeq Reagent Kit v3 for 600 cycles (Illumina). The sequence data obtained in this study were deposited in the DDBJ database under accession numbers SAMD00219124–SAMD00219214.

**Table 1:** Oligonucleotide sequences used in this study.

Name	Sequence (5′- 3′)	Reporter	Quencher	Use	Reference
qSeq-MiFish-U-F	tcgtcggcagcgtcagatgtgtataagagacagNNNNNNNNGYCGGTAAAACTCGTGCCAGC	NA^†^	NA	qSeq	^19^
qSeq-MiFish-U-R	gtctcgtgggctcggagatgtgtataagagacagNNNNNNNNCATAGTGGGGTATCTAATCCCAGTTTG	NA	NA	qSeq	^19^
F2 primer	TCGTCGGCAGCGTCAGAT	NA	NA	qSeq	^19^
HraF	CACCCCAGCAAACCCCTTA	NA	NA	dPCR	^20^
HraR	ACTAGAATAGAGAACAGTAACGCGAGAA	NA	NA	dPCR	^20^
HraP	CCTGTTCGCTTACGCCATTCTACGATCA	FAM	TAMRA	dPCR	^20^
OlaF	TGCCGCCGCAACAGTT	NA	NA	dPCR	^21^
OlaR	GAAAAGTAAGGGTGGAAGGATACTT	NA	NA	dPCR	^21^
OlaP	TCAAACAACCCAACCGGCCTCAA	FAM	TAMRA	dPCR	^21^
ManF	GGGTGTCCTAGCCCTTCTGTT	NA	NA	dPCR	^21^
ManR	GTATGTCGGCGACTAGGGTTCA	NA	NA	dPCR	^21^
ManP	TGCCAATTCTCCACACATC	VIC	MGB:NFQ	dPCR	^21^

Small case letters in sequences indicate regions for index PCR.

^†^Not applicable.

### Data analysis

First, all sequences were assembled and screened by length and quality of reads using the mothur software package (v1.39.5)^22^. The processed sequence reads were classified using the MiFish pipeline (http://mitofish.aori.u-tokyo.ac.jp/mifish/), with the parameters as previously described^23^. Subsequently, the representative sequences of individual operational taxonomic units (OTUs) were extracted using the Usearch program (http://www.drive5.com/usearch/). The random sequence tags (RST) at the end of sequences in the OTUs were counted to quantify the environmental DNA from each fish species as described elsewhere^16^. For comparison, the relative proportion of eDNA from individual species in each sample was calculated from the composition of the sequences of the fish species obtained by qSeq.

### Microfluidic digital PCR

Quantification of eDNA was also performed by microfluidic dPCR using the BioMark Real-time System and 12.765 Digital Array (Fluidigm Corporation, South San Francisco, CA, United States) as previously described^13^. For each sample, the PCR mixture (6 µL) contained 2 × Probe qPCR mix (3.0 µL; Takara Bio Inc.), 20 × binding dye sample loading reagent (0.6 µL; Fluidigm Corporation), forward and reverse primers (900 nM), TaqMan probe (125 nM), ROX solution (0.015 µL), and sample DNA (1.0 µL). We used three sets of primers and probes to quantify the eDNA of *H. neglectus*, *O. latipes*, and *M. anguillicaudatus* (Table 1). PCR was initiated at 98 °C for 2 min, followed by 50 cycles of 98 °C for 10 s and 60 °C for 1 min. The amplification curves obtained from individual reaction chambers of the microfluidic chip were analysed using Fluidigm Digital PCR analysis software (Fluidigm Corporation) to obtain abundance of DNA molecules.

### Statistical analysis

We employed Gaussian Type II regression models with the standardised major axis method to determine the relationship between the log_10_ eDNA abundances obtained from qSeq and dPCR analyses with the “sma” function of the “smatr” ver. 3.4.8 package in R ver. 3.6.0^24^. Zero values were disregarded for the modelling. We employed the Gaussian Type II model because our preliminary evaluation showed higher R^2^ values for Type II regression models with a Gaussian distribution than for those with a logarithmic distribution in all cases. We compared the differences in the coefficient values by overlapping the 95% confidence interval (CI) ranges.

## Results

In this study, the abundance of eDNA (i.e. the fish mitochondrial 12S rRNA gene copy number) in the extracted DNA from the aquarium experiments using two mock fish communities was quantified by dPCR and qSeq. We used qPCR to quantify the eDNA of only three fish species, *Hemigrammocypris neglectus*, *Misgurnus anguillicaudatus*, and *Oryzias latipes*, whereas qSeq was used to quantify the eDNA of all five fish species used in this study. We also calculated the relative abundances of the sequences of each fish species in the sequence library obtained by HTS using MiFish primers for comparison with the dPCR and qSeq results.

**Figure 3:**
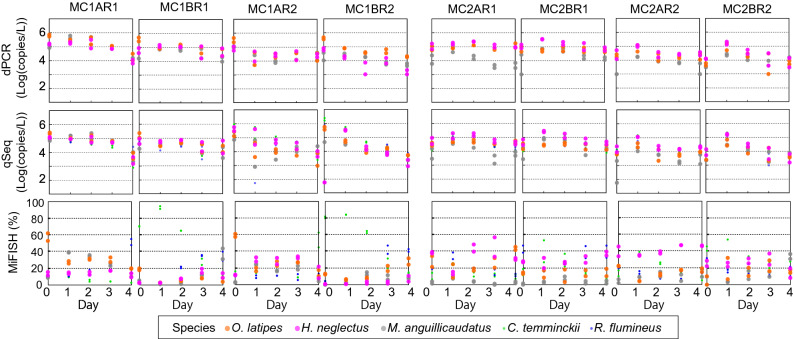
Quantification of eDNA released from 5 fish species in the aquarium experiments. The 12 plots on the left show results from the aquaria using mock fish community 1 while the 12 plots on the right show the results from mock fish community 2.

For MC1, both dPCR and qSeq showed a trend of decreasing abundance of fish eDNA with time. The quantified values by dPCR were 5.0 × 10^3^–7.2 × 10^5^ copies, 1.0 × 10^3^–3.0 × 10^5^ copies, and 4.0 × 10^3^–5.9 × 10^5^ copies/L for *O. latipes*, *H. neglectus*, and *M. anguillicaudatus*, respectively (Fig. 3, left, top row). The abundance of eDNA quantified by qSeq ranged from 9.8 × 10^2^–5.4 × 10^5^ copies, 8.2 × 10^2^–5.8 × 10^5^ copies, and 8.3 × 10^2^–2.5 × 10^5^ copies/L for *O. latipes*, *H. neglectus*, and *M. anguillicaudatus*, respectively (Fig. 3, left, middle row). In addition to these three fish species whose eDNA could be quantified using previously established assays (i.e. with specific primers and probes), qSeq can quantify the eDNA of two further fish species, *Candidia temminckii* and *Rhinogobius flumineus,* without establishing species-specific assays. The latter two species showed eDNA abundances within the range of the other three species, and a similar trend of decreasing eDNA over time.

MC2 included three *H. neglectus* individuals compared with one in MC1. In MC2, the eDNA abundance was consistently highest on day 1 and, the abundance tended to decrease over time after day 1. The quantified values by dPCR were 1.0 × 10^3^–1.7 × 10^5^ copies, 3.0 × 10^3^–3.5 × 10^5^ copies, and 1.0 × 10^3^–1.1 × 10^5^ copies/L for *O. latipes*, *H. neglectus*, and *M. anguillicaudatus*, respectively (Fig. 3, right, top row). The time-series profiles of eDNA abundance obtained by qSeq are consisted with dPCR (Fig. 3, right, middle row) and ranged from 2.1 × 10^3^–6.9 × 10^4^, 1.6 × 10^3^–2.9 × 10^5^, and 2.1 × 10^3^–6.9 × 10^4^ for *O. latipes*, *H. neglectus*, and *M. anguillicaudatus*, respectively. The other two species (*C. temminckii* and *R. flumineus*), which were only quantified using qSeq, showed a similar trend, with eDNA abundances peaking on day 2 and then decreasing over time.

Although the relative proportions obtained in MiFish are not comparable with the absolute quantitative values obtained by dPCR or qSeq, the relative proportions in the MiFish sequence library (Fig. 3, bottom row) followed different trends to the results from the two quantitative methods. In MC1, the MiFish results were similar to the dPCR and qSeq results for AR1 and AR2. However, BR1 and BR2 generated completely different MiFish results; the relative abundance of eDNA increased with time for three of the five fish. More specifically, in MC1BR2, the relative abundance of *H. neglectus* eDNA was 1% on day 0, and increased to 4–11% by day 4. The relative abundances of *O. latipes* and *M. anguillicaudatus* in the sequence library also increased over time. However, these results were concurrent with a decrease in the relative abundance of *C. temminckii* eDNA, and this might create a false impression that the eDNA abundances of other fish species increased over time. Furthermore, the relative abundances in the sequence library were not consistent with the results of the two quantitative methods. For instance, in MC2AR2, the lowest relative abundance was observed on day 2 and the trend in relative abundance of *O. latipes* indicated by the MiFish results was opposite to that inferred with the dPCR and qSeq methods. These results confirm that relative abundances alone cannot be used to quantitatively discuss the abundance of eDNA.

**Figure 4:**
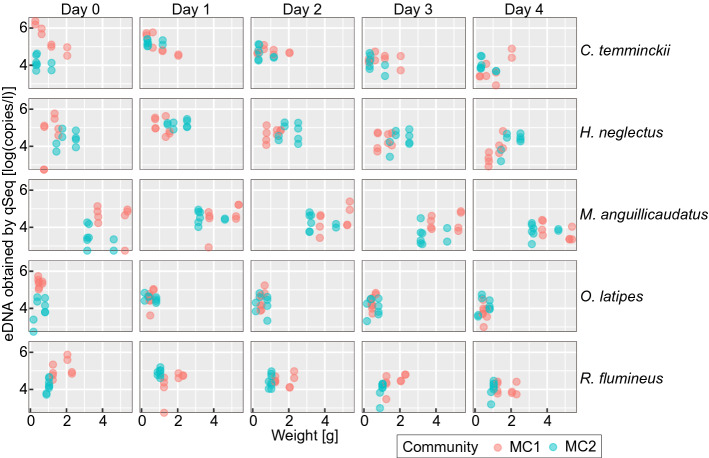
Correlation between biomass (fish weight) and the abundance of eDNA at all five time points quantified by qSeq.

Previous studies have demonstrated a correlation between biomass and the density of eDNA in natural or laboratory environments^25,26^. In this study, however, no significant correlation was observed between biomass and abundance of eDNA (Fig. 4). The mean weight of *M. anguillicaudatus* was 4.2 g, which was approximately tenfold higher than that of *O. latipes* at 0.47 g (Table 2). However, the abundance of eDNA from *M. anguillicaudatus* was generally lower than that from *O. latipes*, regardless of the quantification method (Fig. 3). This discrepancy might be attributed to differences in the discharge rate of eDNA between fish species. MC2 contained three individuals of *H. neglectus* compared with one in MC1; however, the abundance of *H. neglectus* eDNA was lower in MC2 than in MC1 (Fig. 4).

**Table 2:** Composition and weight of the species used in the aquaria.

	^‡^ Weight (g)							
Species	MC1AR1	MC1BR1	MC1AR2	MC1BR2	MC2AR1	MC2BR1	MC2AR2	MC2BR3
*C. temminckii*	1.16	2.04	0.62	0.27	0.36	2.04	0.32	1.17
*H. neglectus*	0.77	1.55	1.34	0.75	1.77 ^†^	2.32 ^†^	2.52 ^†^	1.44 ^†^
*M. anguillicaudatus*	5.38	3.76	3.72	5.24	3.24	4.87	3.15	4.62
*O. latipes*	0.66	0.46	0.48	0.43	0.38	0.38	0.81	0.17
*R. flumineus*	2.30	1.24	1.25	2.05	1.04	2.07	1.03	0.89

^†^Sum of three individuals.

^‡^The weight was measured at the end of the experiment (day 4).

**Figure 5:**
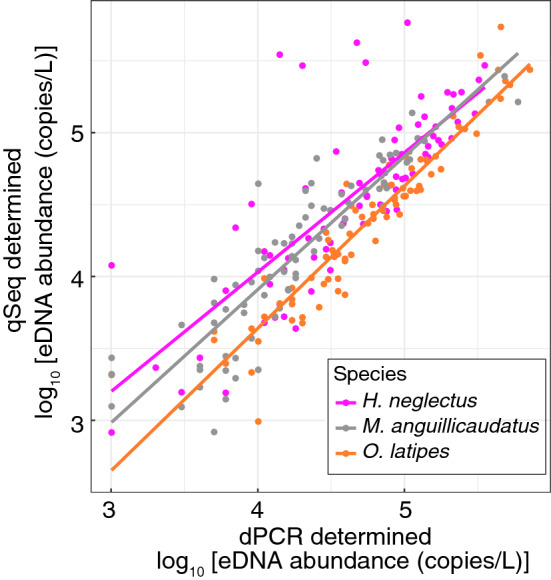
Correlation between eDNA copy number as quantified by digital PCR (dPCR) and quantitative sequencing (qSeq). Lines indicate regressions for each species, where pink is *H. neglectus*: [log(qSeq) = 1.052(dPCR) − 0.043, R^2^ = 0.643, *P* < 0.0001, Gaussian Type II regression]; grey is *M. anguillicaudatus*: [log(qSeq) = 1.114(dPCR) − 0.112, R^2^ = 0.859, *P* < 0.0001]; and orange is *O. latipes*: [log(qSeq) = 1.074(dPCR) − 0.058, R^2^ = 0.786, *P* < 0.0001].

The abundance of eDNA from each fish species quantified by dPCR was strongly correlated with that quantified using qSeq (Fig. 5). The correlations were significant (*P* < 0.001), with R^2^ values of 0.643, 0.859, and 0.786 for *H. neglectus*, *M. anguillicaudatus*, and *O. latipes*, respectively. The relationships between qSeq and dPCR results had slopes of ~ 1 and were not significantly different to 95% CIs. However, the *O. latipes* eDNA abundance values were higher with dPCR than with qSeq for most of the samples. The clear significant linear correlation between quantified abundances obtained by dPCR and qSeq indicates that using qSeq instead of the standard HTS can add quantitative information to species composition data based on obtained sequences without establishing a specific assay for each fish species of interest.

## Discussion

In the present study, we employed the qSeq technique to quantify eDNA in mesocosm experiments, demonstrating that eDNA from 5 fish species can be sequenced and quantified simultaneously by qSeq. The values quantified by dPCR tended to be slightly higher than those obtained by quantitative sequencing (qSeq). These results might be due to non-specific binding of the probes and subsequent digestion, resulting in overestimation by dPCR. Alternatively, the qSeq-MiFish-U-F primer might not have hybridised to all target eDNA during single primer extension, causing underestimation by qSeq. Since the amplification efficiency of PCR is generally around 80–90%^27^, depending on the DNA synthase and amplicon length, this may have led to a slight underestimation of the efficiency of the reaction, even though, in SPE, the reaction mixture was gradually cooled from 94 °C to the annealing temperature (60 °C) over about 5 min and then annealed for 1 min to increase the annealing efficiency. Compared to dPCR, where DNA can be quantified whenever amplification is confirmed in 50 cycles of PCR, quantitative sequencing, where single-stranded DNA must be synthesised in the SPE reaction for quantification, may be more influenced by differences in PCR efficiency.

Among the three fish species for which dPCR and qSeq were compared, the abundance obtained with qSeq was lower than that obtained with dPCR for *O. latipes*, with most of the plots falling below the 1:1 line (Fig. 5). The mtDNA sequence of *O. latipes* has two mismatches to qSeq-MiFish-U-F, may have resulted in lower abundance obtained by qSeq. The primer was designed to be universal for all fishes; however, the amplification of *O. latipes* eDNA may not have been optimal owing to these mismatches. Therefore, improvements such as the introduction of degenerate bases into the primer would be necessary for accurate quantification of *O. latipes* eDNA. In addition, increased stringency of the SPE reactions may be necessary to prevent off-target sequence amplification. Since increasing the annealing temperature is likely to reduce the efficiency of the reaction, it may be preferable to use artificial nucleic acids such as LNA and PNA to increase the annealing specificity^28^. Despite not being the target of the primers, *O. latipes* was detected in both qSeq and the conventional sequencing. The sensitivity this off-target sequence is thought to be higher with qSeq than with the conventional sequencing. This is because in the conventional PCR the regions complementary to the primers in PCR-replicated DNA would not have a mismatch, and so the effect of the primer sequence mismatch diminishes as the PCR proceeds, whereas in qSeq, DNA would not be quantified if the SPE do not occur due to the mismatch. Related to this, conventional qPCR may be more sensitive than qSeq when the number of target DNA is small^15^, as it may be difficult to hybridise all target DNA molecules in one step of SPE.

We found that the abundance of fish eDNA in aquaria was highest just after the fish were added and tended to decrease over time. This is consistent with previous studies that detected the highest abundance of fish eDNA at the start of experiments, and attributed this to the struggles during the acclimatisation of introduced fish to the environment^11,29–32^. However, we found that different mock fish communities had different peaks in eDNA abundance, with MC1 peaking on day 0, and MC2 peaking on day 1. The reason for this is not known, indicating that further studies of eDNA and fish behaviour are needed. The abundances of fish species obtained using HTS were very different from the absolute copy numbers of eDNA quantified with dPCR and qSeq. In general, PCR efficiency can affect the proportions of sequences in sequence libraries. A range of factors known to affect PCR efficiency, such as primer-target mismatches, sequence composition (e.g. GC content), and the bases adjacent to primers^7–10^ may have influenced the proportions of sequences in our fish sequence library, resulting in different proportions compared with the original eDNA samples.

Quantitative sequencing can simultaneously quantify DNA from multiple species by adding a single step to the standard HTS procedure. This eliminates the need to establish specific assays for individual target species, which are required with dPCR or qPCR, that involve the design of primers and probes and optimisation of conditions. While qSeq could quantify eDNA, the aquarium experiments in this study did not show a significant correlation between biomass and abundance of eDNA. Future research using qSeq for comprehensive quantification of eDNA in the natural environment will provide a better understanding of the dynamics of eDNA released from organisms by accumulating data on the relationship between eDNA abundance and population numbers.

## Data Availability

The DNA sequencing data obtained in this study are deposited in the DNA Data Bank of Japan under accession nos SAMD00219124–SAMD00219214, and the datasets generated during the current study are available from the corresponding author on reasonable request.

## References

[1] Barnes MA, Turner CR (2016). The ecology of environmental DNA and implications for conservation genetics. Conserv. Genet..

[2] Thomsen PF, Willerslev E (2015). Environmental DNA—an emerging tool in conservation for monitoring past and present biodiversity. Biol. Conserv..

[3] Deiner K (2017). Environmental DNA metabarcoding: transforming how we survey animal and plant communities. Mol. Ecol..

[4] Tsuji S, Takahara T, Doi H, Shibata N, Yamanaka H (2019). The detection of aquatic macroorganisms using environmental DNA analysis—a review of methods for collection, extraction, and detection. Environ. DNA.

[5] Parducci L (2017). Ancient plant DNA in lake sediments. New Phytol..

[6] Willerslev E (2007). Ancient biomolecules from deep ice cores reveal a forested southern Greenland. Science.

[7] Ben-Dov E, Shapiro OH, Kushmaro A (2012). ‘Next-base’ effect on PCR amplification. Environ. Microbiol. Rep..

[8] Ruijter JM (2009). Amplification efficiency: linking baseline and bias in the analysis of quantitative PCR data. Nucleic Acids Res..

[9] Salipante SJ (2014). Performance comparison of Illumina and ion torrent next-generation sequencing platforms for 16S rRNA-based bacterial community profiling. Appl. Environ. Microbiol..

[10] Sipos R (2007). Effect of primer mismatch, annealing temperature and PCR cycle number on 16S rRNA gene-targetting bacterial community analysis. FEMS Microbiol. Ecol..

[11] Takahara T, Minamoto T, Yamanaka H, Doi H, Kawabata Z (2012). Estimation of fish biomass using environmental DNA. PLoS ONE.

[12] Doi H (2017). Environmental DNA analysis for estimating the abundance and biomass of stream fish. Freshw. Biol..

[13] Hoshino T, Inagaki F (2012). Molecular quantification of environmental DNA using microfluidics and digital PCR. Syst. Appl. Microbiol..

[14] Wilcox TM (2020). Parallel, targeted analysis of environmental samples via high-throughput quantitative PCR. Environ. DNA.

[15] Hoshino T, Inagaki F (2017). Application of stochastic labeling with random-sequence barcodes for simultaneous quantification and sequencing of environmental 16S rRNA genes. PLoS ONE.

[16] Hoshino T, Hamada Y (2017). Estimation of the influence of sequencing errors and distribution of random-sequence tags on quantitative sequencing. J. Biosci. Bioeng..

[17] Fu GK, Hu J, Wang P-H, Fodor SPA (2011). Counting individual DNA molecules by the stochastic attachment of diverse labels. Proc. Natl. Acad. Sci. U. S. A..

[18] Uchii K, Doi H, Minamoto T (2016). A novel environmental DNA approach to quantify the cryptic invasion of non-native genotypes. Mol. Ecol. Res..

[19] Miya M (2015). MiFish, a set of universal PCR primers for metabarcoding environmental DNA from fishes: detection of more than 230 subtropical marine species. R. Soc. Open Sci..

[20] Fukuoka A, Takahara T, Matsumoto M, Ushimaru A, Minamoto T (2016). Establishment of detection system for native rare species, *Hemigrammocypris rasborella*, using environmental DNA. Jpn. J. Ecol..

[21] Jo T, Fukuoka A, Uchida K, Ushimaru A, Minamoto T (2020). Multiplex real-time PCR enables the simultaneous detection of environmental DNA from freshwater fishes: a case study of three exotic and three threatened native fishes in Japan. Biol. Invasions.

[22] Schloss PD (2009). Introducing mothur: open-source, platform-independent, community-supported software for describing and comparing microbial communities. Appl. Environ. Microbiol..

[23] Sato Y, Miya M, Fukunaga T, Sado T, Iwasaki W (2018). MitoFish and MiFish pipeline: a mitochondrial genome database of fish with an analysis pipeline for environmental DNA metabarcoding. Mol. Biol. Evol..

[24] Warton DI, Wright IJ, Falster DS, Westoby M (2006). Bivariate line-fitting methods for allometry. Biol. Rev..

[25] Yates MC, Fraser DJ, Derry AM (2019). Meta-analysis supports further refinement of eDNA for monitoring aquatic species-specific abundance in nature. Environ. DNA.

[26] Doi H (2015). Use of droplet digital PCR for estimation of fish abundance and biomass in environmental DNA surveys. PLoS ONE.

[27] Arezi B, Xing W, Sorge JA, Hogrefe HH (2003). Amplification efficiency of thermostable DNA polymerases. Anal. Biochem..

[28] You Y, Moreira BG, Behlke MA, Owczarzy R (2006). Design of LNA probes that improve mismatch discrimination. Nucleic Acids Res..

[29] Andersen K (2012). Meta-barcoding of ‘dirt’ DNA from soil reflects vertebrate biodiversity. Mol. Ecol..

[30] Barnes M (2014). Environmental conditions influence eDNA persistence in aquatic systems. Environ. Sci. Technol..

[31] Jo T, Murakami H, Yamamoto S, Masuda R, Minamoto T (2019). Effect of water temperature and fish biomass on environmental DNA shedding, degradation, and size distribution. Ecol. Evol..

[32] Sassoubre LM, Yamahara KM, Gardner LD, Block BA, Boehm AB (2016). Quantification of environmental DNA (eDNA) shedding and decay rates for three marine fish. Environ. Sci. Technol..

