# Author Correction: Simultaneous absolute quantification and sequencing of fish environmental DNA in a mesocosm by quantitative sequencing technique

**DOI:** 10.1038/s41598-021-91557-w

**Published:** 2021-06-01

**Authors:** Tatsuhiko Hoshino, Ryohei Nakao, Hideyuki Doi, Toshifumi Minamoto

**Affiliations:** 1 Kochi Institute for Core Sample Research, Japan Agency for Marine-Earth Science and Technology, Kochi, Japan; 2 Graduate School of Science and Technology for Innovation, Yamaguchi University, Yamaguchi, Japan; 3 Graduate School of Human Development and Environment, Kobe University, Kobe, Japan; 4 Graduate School of Simulation Studies, University of Hyogo, Kobe, Japan

Correction to: *Scientific Reports* 10.1038/s41598-021-83318-6, published online 23 February 2021

In this Article, the authors neglected to include a potential conflict of interests. The Competing Interests section should read:

“T.H. reported a potential conflict of interest related to patent royalties of the quantitative sequencing method for eDNA quantification. The other authors declare no competing interests.”

